# Body image satisfaction, sociodemographic, functional and clinical aspects of community-dwelling older adults

**DOI:** 10.1590/1980-57642018dn12-030012

**Published:** 2018

**Authors:** Raquel Rousselet Farias, Renata Breda Martins, Vivian Ulrich, João Henrique Correa Kanan, Irenio Gomes da Silva, Thais de Lima Resende

**Affiliations:** 1 Specialist. School of Health Sciences, Pontifical Catholic University of Rio Grande do Sul, Porto Alegre, RS, Brazil.; 2 Specialist. School of Health Sciences, Pontifical Catholic University of Rio Grande do Sul, Porto Alegre, RS, Brazil.; 3 Specialist. School of Health Sciences, Pontifical Catholic University of Rio Grande do Sul, Porto Alegre, RS, Brazil.; 4 PhD. Department of Microbiology, Immunology and Parasitology, Federal University of Rio Grande do Sul, Porto Alegre, RS, Brazil.; 5 PhD. Institute of Geriatrics and Gerontology Pontifical Catholic University of Rio Grande do Sul, Porto Alegre, RS, Brazil.; 6 PhD. School of Health Sciences, Pontifical Catholic University of Rio Grande do Sul, Porto Alegre, RS, Brazil.

**Keywords:** aged, aging, body image, primary health care, public health, idoso, envelhecimento, imagem corporal, atenção primária à saúde, saúde pública

## Abstract

Body image, according to the definition by Ledoux et al.(1) is “the systematic, cognitive, affective, conscious, and unconscious representation that people have concerning their bodies during their biological development and throughout their social relationships”.

**Objective:**

To determine the prevalence of body image satisfaction (BIS) and its relationship with sociodemographic, functional and clinical aspects in older adults.

**Methods:**

A cross-sectional, analytical and prospective study of a random sample of older adults from all health districts of Porto Alegre (30 health units) was conducted. The following aspects were studied: sociodemographic data (sex, age, marital status and education), BIS (Stunkard’s scale), functional tests (30 seconds Sit/Stand Test, time to walk 10m, Handgrip Strength - HGS), physical activity (Minnesota Questionnaire) and cognition (Mini-Mental State Examination).

**Results:**

Most of the 532 participants were dissatisfied with their body image (92.5%), particularly the women (71.7%). After Binary Logistic Regression (6 steps), BIS predictors were: high scores for the Sit/Stand (OR: 1.13; p=0.013), higher HGS (OR: 1.06; p=0.049), shorter time engaged in physical activity (OR: 0.77; p<0.001).

**Conclusion:**

The prevalence of BIS was low and most of the variables analyzed bore no relation to BIS. Notwithstanding, a relationship was found with greater HGS, higher Sit/Stand score and less time engaged in physical activity. Given the scarcity of studies on this subject, our study furthers the knowledge on how body image affects this population group.

## Resumo

A imagem corporal, de acordo com a definição de Ledoux et al.(1), é “a representação sistemática, cognitiva, afetiva, consciente e inconsciente que as pessoas possuem em relação ao seu corpo durante o seu desenvolvimento biológico e ao longo de suas relações sociais”.

**Objetivo:**

Determinar a prevalência de satisfação com a imagem corporal (BIS) e sua relação com aspectos sociodemográficos, funcionais e clínicos em idosos.

**Métodos:**

Estudo transversal, analítico e prospectivo, realizado em uma amostra aleatória de idosos de todos os distritos sanitários de Porto Alegre (30 unidades de saúde). Os seguintes aspectos foram estudados: dados sociodemográficos (sexo, idade, estado civil e escolaridade), BIS (escala de Stunkard), testes funcionais (30 segundos Sit/Stand Test, tempo de caminhada 10m, Handgrip Strength - HGS) atividade física (Minnesota Questionnaire) e cognição (Mini Mental State Examination).

**Resultados:**

A maioria dos 532 participantes estava insatisfeita com a imagem corporal (92,5%), particularmente as mulheres (71,7%). Após a Regressão Logística Binária (6 passos), os preditores do BIS foram: escores altos para o sentar/levantar (OR: 1,13; p=0,013), maior FP (OR: 1,06; p=0,049), menor tempo gasto em atividade física (OR: 0,77; p<0,001).

**Conclusão:**

A prevalência do BIS foi baixa e a maioria das variáveis ​​analisadas não apresentou relação com o BIS. Não obstante, foi encontrada uma relação com maior FPP, maior escore de Sit/Stand e menor tempo gasto em atividade física. Devido à escassez de estudos sobre o assunto, nossa pesquisa ajuda a aumentar o conhecimento sobre como a imagem corporal afeta essa parcela da população.

Body image, according to the definition by Ledoux et al.^1^ is “*the systematic, cognitive, affective, conscious, and unconscious representation that people have concerning their bodies during their biological development and throughout their social relationships*”. It is still scarcely reported in the literature and most studies evaluate adolescents and adults.^2^^-^ 4 The investigations with younger populations point to the predominance of body image dissatisfaction among women^3^^,^ 5 and its association with the risk of developing chronic diseases,^6^ difficulty in changing habits that are harmful to health,^7^ besides worse quality of life, physical and mental health.^8^

The paucity of studies on body image satisfaction (BIS) is most evident regarding studies with older adult populations and its relationship with other characteristics inherent to the aging process.^2^^,^ 9 As is the case in younger individuals, when body image is analyzed in older adults dissatisfaction is frequent.^3^^,^ 9 ^-^ 14 However, in spite of the higher prevalence of body image dissatisfaction, there are indications that the greatest concern in this age group is physical ability/competence,^2^^,^ 9 ^,^ 15 while BIS is positively associated with perceived good health,^15^ quality of life and well-being.^15^^,^ 16

Inexorable, physiological aging promotes significant losses in the various systems of the human body which, when associated with pathological and/or degenerative processes, can exacerbate them and lead to physical dependence and loss of autonomy.^17^ Thus, within the global and national situation of a rapidly aging population,^18^ as well as the impact this has on health systems,^17^ it is relevant to study this construct in terms of health and quality of life. Moreover, users of public primary care units in Brazil have greater social, environmental and health vulnerability,^19^ which is even greater among the aged population. The objective of the present study, therefore, was to determine the prevalence of BIS and its relationship with sociodemographic, functional and clinical aspects of older public Primary Care users from Porto Alegre, Rio Grande do Sul state, Brazil.

## METHODS

This cross-sectional analytical study was conducted from March 2011 to December 2012 with the approval of the Research Ethics Committee of the Pontifical Catholic University of Rio Grande do Sul - PUCRS (protocol number 10/04967) and the City Health Department of Porto Alegre/RS (001.021434.10.7),^20^ fully complying with the Declaration of Helsinki. Written informed consent forms were signed by each participant or carer. A detailed description of all methods adopted for the present study can be found in Gomes et al.^20^

The study involved a random sample of 587 older adults from 30 different public Primary Health Care units (Family Health Strategy units) in Porto Alegre, representative of all the city’s health districts. This particular set of health units - Family Health Strategy - are usually established in areas that have greater social, environmental and health vulnerability.^19^

The study was run in two phases: [1] General data collection carried out at subjects’ homes, where an instrument was used to collect sociodemographic and economic data (Older Adult Global Assessment Questionnaire), as well as reported morbidity and behaviors that interfere with health; [2] Specialized evaluations carried out at the São Lucas Hospital of the PUCRS by a multidisciplinary team assessing the clinical, nutritional and physical aspects of the older adults.^20^

The study included people aged 60 years and over, who were registered users of the 30 randomly selected health units. Those who were unable to travel to the outpatient clinics of the São Lucas Hospital for the multiprofessional evaluation using the transportation offered by the research group were excluded.^20^

For the present study, the following variables were used: gender, age, age group, marital status, education, BIS (Stunkard’s Scale),^21^ the 30s Sit/Stand test,^22^ handgrip strength (HGS),^23^ time (seconds) to walk 10 meters,^24^ the number of minutes per week engaged in light, moderate and intense physical activity,^25^^,^ 26 the presence or absence of cognitive decline (Mini-Mental State Examination),^27^ urinary incontinence, reported falls in past year, number of falls in past year, fracture due to falls, use of medication, and polypharmacy. The description of the variables, the instruments used, and the place of data collection are shown in detail in Table 1.

**Table 1:** Description of study variables, instruments used and place of data collection.

Variable type	Variable	Categories	Data collection	
			Instrument	Place
Sociodemographic	Age	None	OAGAQ*	Home
	Age group	60-69; 70-79; ≥80years	OAGAQ*	Home
	Education	Illiterate; low; medium; high education	OAGAQ*	Home
	Marital status	Living with a partner/not living with a partner	OAGAQ*	Home
Functional	Time (s) to walk 10m	None	Chronometer	SLH+
	Sit/Stand Test (repetitions)	None	Chronometer	SLH+
	Handgrip strength (Kgf)	None	Dynamometer Crow	SLH+
	Physical activity level (min/week)	Total; light; moderate; intense	Minnesota Questionnaire	SLH+
Clinical	Cognition	None	MMSE§	SLH+
	Presence of cognitive loss	Yes/No	MMSE§	SLH+
	Body image satisfaction	Yes/No	Stunkard’s Scale	SLH+
	Urinary incontinence	Yes/No	OAGAQ*	SLH+
	Report of falls in the last year	Yes/No	OAGAQ*	SLH+
	Nº of falls in the past year	1; 2; ≥3	OAGAQ*	SLH+
	Fracture due to fall	Yes/No	OAGAQ*	SLH+
	Medication use	Yes/No	OAGAQ*	SLH+
	Nº of medicines taken	None; 1-3; ≥4	OAGAQ*	SLH+

* OAGAQ: Older Adult Global Assessment Questionnaire;

+ SLH: São Lucas Hospital of the Pontifical Catholic University of Rio Grande do Sul;

§ MMSE: Mini-Mental State Examination.

Data were analyzed using the Statistical Package for Social Sciences version 20.0 (SPSS Inc., Chicago, IL). A 5% significance level was used for statistical decisions. The data distribution study was performed using the Kolmogorov-Smirnov test.

For the bivariate analyzes, the following tests were used: Student’s *t*, Mann Whitney U and Pearson’s Chi-square with continuity correction. For the estimation of the measure of effect, the Odds Ratio (OR) was used, with a 95% confidence interval (95% CI).

As the “Body Image Satisfaction” outcome had a binary distribution, Logistic Regression was applied, where all variables with values of p<0.200 in the bivariate analysis were selected for the multivariate analysis. The Backward (conditional) method was used from the saturated model. The association was evaluated using the likelihood-ratio test (2 LL or -2log) while the Nagelkerke and Hosmer-Lemeshow R² estimators were employed to evaluate the quality of fit of final logistic regression model. The probability of gradual entry of the variables into the model was 0.05 and for removal was 0.10. At the cut-off point, the significance was 0.50 for the maximum of 20 interactions. Levels below 0.01 were considered significant, based on the Bonferroni criterion.

## RESULTS

Initially comprising 587 older adults; the final sample had 532 cases, after the exclusion of 55 cases (9.4%) due to missing data for the variable “Satisfaction with body image”. The sample (Table 2) contained predominantly individuals that were women (64.8%), single (71.9%), illiterate (92.8%), and with a significantly higher mean age (69.4±7.5 years) than men (68.2±6.5 years).

**Table 2:** Sociodemographic characteristics and physical activity of sample, according to body image satisfaction.

Variables		Body image satisfaction		p*	Crude OR+
		Yes (n=165)	No (n=367)		95% CI§
Sex||	Male	83 (50.3)	104 (28.3)	<0.001**	2.560 (1.750-3.744)
	Female	82 (49.7)	263 (71.7)	1.0	
Age (years)	Mean±SD¶ (Range)	69.3±7.9 (60.0-103.8)	68.7±6.8 (58.9-95)	0.402++	1.011 (0.9866-1.037)
Age group||	60-69 years	103 (62.4)	235 (64.0)	0.840**	1.0
	70-79 years	47 (28.5)	104 (28.3)		1.002 (0.681-1.162)
	≥80 years	15 (9.1)	28 (7.6)		1.056 (0.841-1.155)
Marital status||	Living with a partner	70 (42.4)	135 (36.8)	0.216**	1.266 (0.871-1.842)
	Not living with a partner	95 (57.6)	232 (63.2)		1.0
Education||	Illiterate	147 (89.1)	339 (92.4)	0.213**	1.0
	Low Education	18 (10.9)	28 (7.6)		0.675 (0.362-1.258)
Total Physical Activity (min/week)§§ Median (1^st^-3^rd^ Quartile)		2,519.0 (1,500.0-4,917.0)	3,420.0 (1,831.0-4,341.0)	0.067||-||	0.811 (0.599-1.033)
Light Physical Activity (min/week)§§ Median (1^st^-3^rd^ Quartile)		1,866.0 (902.0-3,122.0)	2,382.0 (1,384.0-4,341.0)	0.078||-||	0.855 (0.602-1.016)
Moderate Physical Activity (min/week)§§ Median (1^st^-3^rd^ Quartile)		1,429.0 (598.0-2,721.0)	1,541.0 (530.0-3,409.0)	0.081||-||	0.892 (0.577-1.098)
Intense Physical Activity (min/week)§§ Median (1^st^-3^rd^ Quartile)		464.0 (280.0-1,276.0)	873.0 (285.0-2,078.5)	0.330||-||	0.694 (0.388-1.106)

* Minimum level of significance for bivariate analysis;

+ Odds Ratio;

§ 95% confidence interval for OR;

|| Data expressed as n (%);

¶ Standard deviation;

** Pearson’s Chi-square;

++ Student’s *t*-test for independent groups;

§§ Physical activity measured with Minnesota Questionnaire;

||-|| Mann Whitney test.

Men were 2.5 times more likely to be satisfied with their body image than women (Table 2), where the majority of the latter were dissatisfied (71.7%). The other variables shown in Table 2 had no significant association with BIS: age, age group, marital status, education and Physical Activity expressed as the four Minnesota Questionnaire scores.

None of the clinical characteristics of the sample were significantly associated with BIS: medicine use, polypharmacy, reported urinary incontinence, presence of cognitive decline, reported falls and fractures in the past year (Table 3).

**Table 3:** Clinical and functional characteristics of sample according to body image satisfaction.

Variables		Body image satisfaction		p*	Crude OR+
		Yes (n=165)	No (n=367)		95% CI§
Medication||	Yes	140 (88.6)	324 (90.5)	0,510§	1.0
	No	18 (11.4)	34 (9.5)		1.068 (0.869-1.313)
Polypharmacy||	Yes	84 (52.2)	208 (58.6)	0.173§	1.0
	No	77 (47.8)	147 (41.4)		1.142 (1.021-1.278)
Urinary incontinence||	Yes	51 (31.5)	120 (33.3)	0.677§	0.943 (0.618-1.367)
	No	111 (68.5)	240 (66.7)		1.0
Presence of cognitive impairment||	Yes	43 (27.6)	109 (30.8)	0.463§	0.855 (0.563-1.298)
	No	113 (72.4)	245 (69.2)		1.0
Fall in past year||	Yes	53 (32.3)	115 (31.4)	0.838§	1.0
	No	111 (67.7)	251 (68.6)		1.042 (0.702-1.546)
Nº of falls in past year||	1	25 (47.2)	46 (41.1)	0.515§	0.793 (0.369-1.702)
	2	16 (30.2)	31 (27.7)		0.716 (0.502-1.195)
	≥3	12 (22.6)	35 (31.3)	1.0	
Fracture after fall||	Yes	12 (22.6)	31 (27.0)	0.551§	0.793 (0.369-1.702)
	No	41 (77.4)	84 (73.0)		1.0
Time to walk 10m (s) Mean±SD¶ (Range)		6.3±1.7 (3.0-13.0)	6.8± (3.0-20.0)	0.104**	0.850 (0.761-1.250)
Sit/Stand Test (number of repetitions/30s) Mean±SD¶ (Range)		9.7±3.4 (1.0-23.0)	8.4±2.9 (1.0-18.0)	0.009**	1.147 (1.076-1.222)
Handgrip Strength (Kgf) Mean±SD¶ (Range)		29.4±9.5 (11.4-50.0)	24.7±8.8 (5.2-45.8)	<0.001**	1.055 (1.033-1.078)

* Minimum level of significance for bivariate analysis;

+ Odds Ratio;

§ 95% confidence interval for Odds Ratio;

§ Pearson’s Chi-square;

|| Data expressed as n (%);

¶ Standard deviation;

** Student’s *t*-test for independent groups.

Regarding functional characteristics (Table 3), greater lower limb muscle strength was significantly associated with BIS where those with higher scores on the Sit/Stand test and greater HGS were 1.15 and 1.06 times more likely to be satisfied, respectively. On the other hand, the time to walk 10m was not significantly associated with BIS (Table 3).

The initial and final models (6 steps) of the Multivariate Binary Logistic Regression applied for the prediction of BIS are shown in Table 4. The initial model included the variables sex, polypharmacy, time to walk 10m, Sit/Stand test, HGS, total minutes per week engaged in light and moderate physical activity. It was decided to use the total minutes per week engaged in physical activity because this variable not only had the minimum level of compatible significance, but also a larger number of valid cases to be analyzed.

**Table 4:** Multivariate Binary Logistic Regression Models for prediction of satisfaction with body image.

Independent variables		Regression coefficient		Sig.||	Adjusted Odds Ratio		
					Exp (B)¶	95% CI*	
		B_crude_ +	SE§			Lower	Upper
Initial model	Male	–0.317	0.453	0.484	0.728	0.300	1.769
	Time to walk 10m	0.060	0.094	0.525	1.061	0.883	1.275
	Sit/Stand	0.124	0.053	0.018	1.132	1.021	1.255
	Handgrip strength	0.073	0.026	0.005	1.076	1.022	1.132
	Total physical activity**	–0.084	0.054	0.050	0.851	0.693	1.074
	No polypharmacy	–0.493	0.404	0.223	0.611	0.277	1.349
	No history of falls	0.009	0.314	0.977	1.009	0.545	1.868
Final model	Sit/Stand	0.116	0.047	0.013	1.123	1.025	1.231
	Handgrip strength	0.060	0.016	0.000	1.062	1.028	1.096
	Total physical activity**	–0.844	0.022	0.004	0.766	0.522	0.908

* Odds Ratio 95% confidence Interval;

+ Crude regression coefficient;

§ Standard error for regression coefficient;

|| Minimum level of significance for regression coefficient;

¶ Odds ratio;

** Physical activity measured with Minnesota Questionnaire in minutes per week.

**Initial Model** – Note: Nagelkerke R^2^ 0.324; Test of Hosmer-Lemeshow (Chi-squared=32.447; p=0.336); Cox & Snell: 0.362; Overall score ratio – confounding matrix: 74.7%. **Final Model** – Note: Nagelkerke R2 0.388; Test of Hosmer-Lemeshow (Chi-squared=46.503; p=0.580); Cox & Snell: 0.362; Overall score ratio – confounding matrix: 84.2%.

In the confounding matrix, the total of correct answers was 75.0%, where the model correctly classified 92.5% of the cases for which satisfaction was negative (dissatisfaction with body image) and 28.6% of the cases that confirmed BIS.

Due to the loss of the predictive power of certain variables, the final model comprised three variables, whereby the individuals satisfied with their body image can be predicted by higher scores for the Sit/Stand test (OR: 1.12; p=0.013); greater HGS (OR: 1.06, p=0.049), less time engaged in physical activity (OR: 0.77; p<0.001).

## DISCUSSION

Less time engaged in physical activity (Table 2), increased muscle strength of upper (HGS) and lower limbs (Sit/Stand) (Table 3) were the only characteristics considered predictors of BIS, among the many variables analyzed in this sample of underprivileged older adults.

We found few reports in the literature similar to the present article. There are scant articles on BIS in this age group while studies associating body image with physical function are even scarcer. There are exceptions, however, such as a study carried out in Spain with institutionalized older adults, which corroborates our findings regarding the relationship between functional tests and BIS. The authors reported a positive relationship between BIS and walking speed, where faster walkers had greater satisfaction with their body image and better perceived health.^14^

Another study also analyzing physical function was conducted by Reboussin et al.,^16^ who found no relationship between exercise tests and body function. They observed that perceived well-being was more related to body function than appearance, suggesting that, unlike younger populations, older adults place more importance on physical function. The authors concluded that there was a distinction between satisfaction with function and satisfaction with appearance among the older adults.^16^

In contrast with our findings, Umstattd et al.^28^ reported that, among older adults, being satisfied with body image was related to higher levels of moderate to intense physical activity. While their sample comprised mostly people with medium-to-high educational level (91.74%),^28^ our study participants were mostly low educated or illiterate. This may explain the conflicting findings, given that low educated older adults tend to be less active and more satisfied with their body weight than individuals who are active and/or partially active with medium or high education,^29^ and also often lack a clear notion of the risk that excess weight can pose.^30^

Moreover, Sarabia^14^ used the Stunkard’s Scale in his sample, also composed mainly of older adults with low education. However, it is not possible to compare their sample’s levels of physical activity against the present sample because we measured and classified the engagement in physical activity using an instrument, while Sarabia^14^ only asked their subjects whether or not they engaged in physical activity (active x inactive). Considering that their subjects were all institutionalized, their level of physical activity was very likely closer to that of the participants of the present study who reported engaging less time in physical activity and declared being satisfied with their body image.

In older adults, the relationship between BIS and longer time engaged in light physical activity might be due to the fact that they place more importance on body function than body appearence.^31^ Therefore, if satisfied with their physical function, they do not see the need to perform more intense physical activity.

As in the general population, there was a predominance of females in the present study sample, where women also made up the majority of individuals dissatisfied with their body image (72.4%). This finding is corroborated by most articles published on this subject,^4^^,^ 6 ^,^ 10 ^,^ 12 ^,^ 16 ^,^ 28 ^,^ 31 ^,^ 32 and there is evidence that this dissatisfaction with their body is something that accompanies women from a young to an old age, as pointed out by longitudinal studies.^4^^,^ 10 ^,^ 12 This lifelong dissatisfaction is likely a result of the pressure that society places upon women through the imposition of ideals regarding body and beauty, which prove unattainable for most women.^33^ Mellor et al.^31^ argued that one of the reasons men are more satisfied with their body image is that they are more realistic with their own body appearance.

In spite of body image dissatisfaction being a predominantly female characteristic, it is not a consensus in the literature, since there are studies that point to the opposite among older adults.^11^^,^ 34 Paxton and Phythian^11^ concluded that men are more affected by age in relation to their BIS and self-esteem, as they feel less attractive with aging. Baker and Gringart^34^ came to a similar conclusion in their study, after demonstrating that men’s self-esteem diminishes greatly as they age.

In contrast with the present study results, another study also conducted in Brazil and using the Stunkard Scale, Menezes et al.^35^ found a larger proportion of older people satisfied with their body image: 51.3% of the women and 68% of the men. These findings contradict those of the vast majority of articles found in the literature, including the present study.^9^^,^ 14 ^,^ 28 ^,^ 36 The authors concluded that being an older man acts as a protective aspect regarding BIS.^35^

We found two main differences between the present study and that by Menezes et al.,^35^ both involving Brazilian older adults and using the Stunkard Scale. While their study was based on the census distribution, which encompasses people from all socioeconomic and demographic strata, the present study sample was drawn from a low-income and poorly educated population that was dependent on public health care, which is offered in areas of greater social vulnerability.^19^ Another explanation for this discrepancy between these two studies of Brazilian older adults may be the climatic, geographical and cultural differences between the two regions where they were carried out, having been conducted in the Northeast in the case of Menezes and in the South for the present study. As observed among younger Brazilians by Alvarenga et al.,^37^ those in the southern region regarded themselves as burlier compared with young people from other regions of the country.

Other authors have reported that BIS in older adults is more related to functionality.^11^^,^ 15 ^,^ 16 ^,^ 28 ^,^ 38 However, these studies were based only on the participants’ self-reports, without aspects of their functioning being measured, as was done in the present study. This methodological difference makes it difficult to compare the findings of the present study with those based only on the reports of the older adults surveyed, since it is known that they tend to overestimate their capacity, which may affect the final result of the studies that use self-report as a way of measuring aspects of functioning in this population group.^39^^,^ 40

Regardless of this possible methodological drawback in some of the studies, there are indications that functioning is a protective factor against body image dissatisfaction.^9^ Umstattd et al.^28^ demonstrated an association between high body function satisfaction and high levels of functional capacity, rather than with physical appearance. Alleva et al.^38^ suggested that, as appearance becomes less important in mature women, other aspects such as functioning become more important. Paxton and Phytian^11^ argued that health and functioning are strong predictors of self-esteem in older adults, in both women and men. Thus, it seems, despite the fact that body image dissatisfaction affects older adults, its meaning is not the same as for young people, since their concern with their body is more related to functional than esthetic aspects.^31^^,^ 35 Moreover, dissatisfaction is often associated with chronic diseases, such as arthritis and cancer.^9^

For older adults with chronic diseases, this body image dissatisfaction is even more of an issue than the classic signs of aging, such as wrinkles, white hair or sagging skin.^41^ This is explained by the fact that loss of functional capacity and health negatively impacts physical appearance, promoting a greater degree of physical discomfort and reduced physical functioning.^36^ This concern of older adults with their body involves something much more complex. It has been suggested that this aspect be followed more closely by health professionals^4^^,^ 9 ^,^ 12 ^,^ 34 since this age group has the greatest risk of developing chronic diseases.

Therefore, it follows that one of the main contributions of the present study to the practice of health professionals in primary care was to demonstrate the feasibility of applying the Stunkard Scale at this level of healthcare, specifically with the population in their care. The instrument is low-cost, readily available, easy and quick to use, independent of educational level or full cognitive function, its application does not need specific training while its interpretation does not involve complicated calculations. Consequently, it should be easy to detect dissatisfied individuals and investigate other relevant aspects related to their health known to be associated with body image dissatisfaction, namely depression, anxiety, eating disorders, concerns about the effects of loss of autonomy or even the impact that the effects of aging can have on their social relationships.^8^ This is particularly relevant to elderly women with lower muscle strength, representing the majority of those dissatisfied with their body image, who may need to be referred for psychological support or programs tailored to help them accept the aging process and their body image.

The present study has several limitations related to its cross-sectional design, with only one measurement point, precluding the establishment of the direction of the causal relationship between BIS and predictive factors found, since this association can also be bidirectional. In spite of its limitations, it is pertinent to emphasize the representativeness of the sample investigated, which was sufficiently large for a prevalence study and selected in a random and proportional manner from all of the city´s health districts, a factor which may have reduced the bias of selection. It is also noteworthy that the determination of the levels of physical activity and the functional tests were applied by trained professionals in the same place and setting for the entire sample, reducing the chances of errors derived from self-reports by the elders, their caregivers or family members.^39^^,^ 40

Therefore, it can be concluded that the prevalence of BIS was low and associated with greater muscle strength and less time engaged in physical activity. Furthermore, as seen in younger populations, men are more satisfied than women with their body image in the elderly population.
